# Host genetic architecture and the landscape of microbiome composition: humans weigh in

**DOI:** 10.1186/s13059-015-0775-1

**Published:** 2015-09-22

**Authors:** Andrew K Benson

**Affiliations:** Department of Food Science and Technology, University of Nebraska, Food Innovation Center, North 21 Street, Lincoln, Nebraska 68588-6205 USA

## Abstract

Comparative analyses of the control of mammalian microbiomes by host genetic architecture reveal striking conserved features that have implications for the evolution of host–microbiome interactions.

See related Research article: http://www.genomebiology.com/2015/16/1/191

## Introduction

Genetic and environmental factors converge in many different ways and bring about remarkable diversity in complex traits. Our growing understanding of the role of the microbiome in complex diseases naturally leads us to examine the concept of the microbiome as a complex trait, and hence to measure genetic versus environmental control of microbiome composition. Quantitative genetic analyses in mouse models, which allow many environmental factors to be measured or controlled, have established that genetic variation at a number of loci affects compositional features of the gut microbiome, supporting our view of the microbiome as a complex, polygenic trait [1–3]. A study by Ran Blekhman and colleagues [4], published recently in *Genome Biology*, is the first to use genome-wide analyses in a human study population to attribute variation in microbiome composition to genetic variation at specific loci in the host genome.

## Human genetic factors and complex traits

Accurate dissection of genetic and environmental contributions to the control of complex traits in human study populations is a holy grail of complex trait research. This goal is elusive because of the confounding effects of environmental factors that cannot be controlled or estimated. Indeed, initial studies of microbiome composition in a large cohort of obese and lean twin pairs that relied on relatively crude statistical analyses showed only a trend toward greater differences between the microbiomes of dizygotic twin pairs than between those of monozygotic pairs (but no statistically significant difference) [5]. Analysis of data from a twin study in the U.K. using a more sophisticated approach to estimate the heritability of each taxon later detected measurable heritability and, hence, a significant contribution of host genetic variation [6]. Thus, the stage was set for an initial attempt at genome-wide associations.

## First glimpse of the genetic architecture of microbiome control in humans

Blekhman et al. [4] have developed a sophisticated analysis to pull out associations from genome-wide scans of two different populations. Their analysis focused initially on a subset of subjects from the Human Microbiome Project (HMP), in which genotypes were inferred from a large number of high-quality single nucleotide polymorphisms (SNPs) gleaned from human DNA that contaminated stool metagenomic data. They found that genetic variation in the host has a measurable effect on diversity in the microbiome. Furthermore, the loci that made measurable contributions are linked to loci for disease susceptibility and for innate and adaptive immune functions. The authors subsequently tested a second population, the UK Twins cohort, for genome-wide associations between host genetics and microbiome diversity using genotype data obtained directly from each of the subjects, and obtained very similar results. Of the 83 significant host loci identified from the HMP population, there were several instances in which variation affects the abundances of multiple taxa from microbiomes across different body sites. Once again, innate and adaptive immunity genes and disease susceptibility genes were involved.

## Conserved features of genetic architecture and microbiome landscapes

The methodologies used by Blekhman and colleagues are an elegant display of the power of bioinformatics and quantitative genomics. Equally exciting is the opportunity for these techniques to be used in comparative studies to reveal a broad understanding of mammalian genetic architectures that affect the compositional landscape of the microbiome. One of the first observations from such comparative studies is that the genetic architecture revealed by any single study is highly context-dependent and only a small number of associations are replicated across studies. The context-dependency stems from the relatively strong contributions of environmental and stochastic factors that affect microbiome composition. Despite the context-dependency, four general features of genetic architecture that influence microbiome composition are shared between humans and mice, and these features merit discussion because they shed light on the very nature of host–microbiome interactions and their evolutionary relationships.

### Host genetic variation affects microbial taxa across the phylogenetic space of the microbiome

This feature was consistently observed in mouse studies of the gut microbiota, and data from the human populations reported by Blekhman et al. [4] extend this feature across multiple body sites. Phylogenetic signal in the microbiota generally reflects the phylogeny of the host [7, 8], and though some of this shared signal may be attributed to the host's ability to adapt to the microbiome, we would expect the majority to arise from the influence of genetic diversity within the host species in constraining the primary framework of the microbiome. Admittedly, such constraint manifests largely through other complex traits such as dietary preference, physiology, and behavioral traits. Nevertheless, the genetic signal seems likely to play direct roles in the constraint because many of the microbial taxa that show the highest heritability form important hubs in co-occurrence networks [6]. This suggests that the taxa that are under control do indeed play important roles in configuring the microbiome, perhaps as keystone taxa. This feature will continue to intrigue us as a comprehensive list of quantitative trait loci (QTLs) and traits begins to emerge from future studies.

### Effect size of a given microbiome QTL is relatively small

In general, the effect sizes of the associations account for less than 10 % of the phenotypic variation, with the largest effect size reported for any microbiome QTL being 19 %. This general feature teaches us that the cumulative effect of genetic variation on a number of taxa, rather than large effects from a small number of loci on a few taxa, is the main driver of microbiome composition. This feature is likely to explain why factors such as diet can overcome genetic influence [2, 9]. From an evolutionary perspective, the importance of cumulative small effects might suggest that the influence of host genetic diversity on the microbiome could be more of a 'bystander effect' than an 'intended target'. A 'bystander effect' might be achieved if pathogens were the primary drivers of allele frequencies at microbiome QTLs, a possibility pointed out by Blekhman et al. [4]. It is worth noting that there is some overlap between QTLs that control susceptibility and resistance to pathogens and microbiome QTLs, but the sample size of pathogens for which such data are available is too small to allow a definitive conclusion.

### Pleiotropy is common among microbiome QTLs

In each of the studies, multiple instances are observed in which QTL peaks for different taxa of the microbiome overlap, implying that variation at a single genomic region influences several different microorganisms, some of which are phylogenetically diverse. In several instances, genes that are involved in innate immunity underlie these pleiotropic QTL peaks, providing a mechanistic basis for how such pleiotropy could be mediated. Blekhman et al. [4] extend this observation further, showing evidence that these genes are enriched and evolving at a higher rate than expected. From an evolutionary standpoint, pleiotropy is generally believed to constrain the rate at which a gene evolves. This traditional view is, however, being challenged, with alternative splicing, structural modularity, and mutable domains being offered as mechanisms that could overcome such constraint [10].

### Pleiotropy is commonly observed among complex disease phenotypes and microbiome phenotypes

**Fig. 1:**
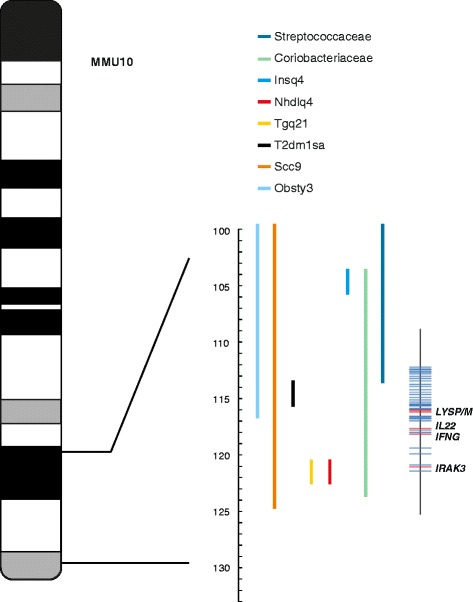
Apparent pleiotropy on mouse chromosome 10. An ideogram of the mouse chromosome 10 is shown with a 100–125 Mb region expanded to the right. The confidence intervals for different quantitative trait loci (QTLs) are indicated by colored solid lines. The QTLs affect both divergent microbial taxa (members of the bacterial families Coriobacteriaceae and Streptococcaceae) and complex diseases, including obesity (Obsty 3 (Obesity QTL3), Nhdlq4 (non-HDL QTL4) and Tgq21 (Triglyceride QTL21)), diabetes (Insq4 (Insulin sensitivity QTL4) and T2dm1sa (Type 2 diabetes mellitus 1)), and colonic tumor susceptibility (scc9). The position of genes associated with inflammation and innate immunity (IL22, IRAK3) and the two primary bacterial cell wall-degrading enzymes (LysP/M) are indicated on the far right by *red bars*

Perhaps the most intriguing conserved feature of the architecture is the consistent finding of overlapping QTLs that are associated with both the control of the microbiome and predisposition to complex diseases or physiological traits related to complex disease. Fig. 1 illustrates an example of one such region, on chromosome 10 of the mouse genome, which was originally detected by Benson et al. [1]. This intriguing feature underscores the role of dysbiosis in complex diseases and, more importantly, it illuminates the concept that genetic predisposition to such diseases occurs, in part, indirectly through imbalance in the microbiome [1]. With the evidence from Blekhman et al. [4] showing that genes that are associated with microbiome QTLs are often under positive selection, such pleiotropy, if real, further implies that balancing selection associated with the microbiome maintains variation in the human population that may elevate the likelihood of disease predisposition.

## Concluding remarks

Clearly, there is much to learn about host–microbiome interactions and their relationships with human and animal disease. For now, humans have 'weighed-in’ among the models in which genome–environment interactions and the microbiome are being studied, and these studies will provide tremendous insights into the mechanistic details of diseases as well as their distributions across populations.

## Abbreviation

QTL: Quantitative trait locus
